# Iodate respiration by *Azoarcus* sp. DN11 and its potential use for removal of radioiodine from contaminated aquifers

**DOI:** 10.3389/fmicb.2023.1162788

**Published:** 2023-04-17

**Authors:** Seiya Sasamura, Toshihiko Ohnuki, Naofumi Kozai, Seigo Amachi

**Affiliations:** ^1^Graduate School of Horticulture, Chiba University, Chiba, Japan; ^2^Fukushima Reconstruction and Revitalization Unit, Institute of Innovative Research, Tokyo Institute of Technology, Tokyo, Japan; ^3^School of Resource Environment and Safety Engineering, University of South China, Hengyang, Hunan, China; ^4^Advanced Science Research Center, Japan Atomic Energy Agency, Ibaraki, Naka-gun, Japan

**Keywords:** iodate respiration, *Azoarcus* sp. DN11, IdrABP_1_P_2_, radioiodine, ^129^I

## Abstract

*Azoarcus* sp. DN11 was previously isolated from gasoline-contaminated groundwater as an anaerobic benzene-degrading bacterium. Genome analysis of strain DN11 revealed that it contained a putative *idr* gene cluster (*idrABP_1_P_2_*), which was recently found to be involved in bacterial iodate (IO_3_^−^) respiration. In this study, we determined if strain DN11 performed iodate respiration and assessed its potential use to remove and sequester radioactive iodine (^129^I) from subsurface contaminated aquifers. Strain DN11 coupled acetate oxidation to iodate reduction and grew anaerobically with iodate as the sole electron acceptor. The respiratory iodate reductase (Idr) activity of strain DN11 was visualized on non-denaturing gel electrophoresis, and liquid chromatography–tandem mass spectrometry analysis of the active band suggested the involvement of IdrA, IdrP_1_, and IdrP_2_ in iodate respiration. The transcriptomic analysis also showed that *idrA*, *idrP_1_*, and *idrP_2_* expression was upregulated under iodate-respiring conditions. After the growth of strain DN11 on iodate, silver-impregnated zeolite was added to the spent medium to remove iodide from the aqueous phase. In the presence of 200 μM iodate as the electron acceptor, more than 98% of iodine was successfully removed from the aqueous phase. These results suggest that strain DN11 is potentially helpful for bioaugmentation of ^129^I-contaminated subsurface aquifers.

## Introduction

Iodine is an essential trace element in humans and animals because it is a constituent of the thyroid hormones triiodothyronine (T_3_) and thyroxine (T_4_) (Dijck-Brouwer et al., 2022). Insufficient dietary iodine can cause deficiency disorders such as endemic goiter and congenital hypothyroidism (Chaker et al., 2022). Iodine deficiency often occurs in inland and mountainous regions, whereas it is less likely to occur in coastal areas where people consume iodine-rich seafood.

Iodine is primarily present in the ocean, and the average concentration of dissolved iodine in seawater is 0.45 μM (Wong, 1991; Fuge and Johnson, 2015). Inorganic iodate (IO_3_^−^) and iodide (I^−^) ions are the predominant chemical forms of iodine in seawater. Thermodynamically, the concentration ratio of iodate to iodide (IO_3_^−^/I^−^) in oxygenated seawater is 10^13.5^ (Sillen, 1961). This indicates that iodate is relatively more stable and that iodide should not be detectable in seawater. However, significant quantities of iodide are observed in certain surface waters (Wong, 1991). This apparent disequilibrium may be due to the biological reduction of iodate to iodide. Generally, it is difficult to convert iodate to volatile iodine species such as I_2_ and CH_3_I. However, iodide is easily converted to I_2_ and CH_3_I and released into the atmosphere (Amachi et al., 2001, 2005). Gaseous iodine compounds emitted from seawater are photolyzed and reach terrestrial environments through wet (rainwater) or dry deposition, where they accumulate in plants and animals (Fuge and Johnson, 2015). Therefore, iodate reduction in the ocean is vital in providing this essential trace element to humans and animals. Gaseous iodine compounds in the atmosphere also contribute to ozone (O_3_) destruction in the troposphere and the formation of cloud condensation nuclei and are thus involved in climate change on a global scale (O'Dowd et al., 2002; Carpenter et al., 2021).

Biological iodate reduction has been studied in marine microalgae (phytoplankton) and bacteria (Amachi, 2008; Yeager et al., 2017). Among these organisms, the molecular mechanism of iodate reduction has recently become clear in dissimilatory iodate-reducing bacteria (DIRB), such as *Pseudomonas* sp. SCT and *Denitromonas* sp. IR-12 (Yamazaki et al., 2020; Reyes-Umana et al., 2022a). DIRB possesses an *idrABP_1_P_2_* gene cluster in its genome. The *idrA* and *idrB* genes encode the large catalytic subunit and electron-transferring small subunit of novel dimethylsulfoxide (DMSO) reductase family protein, Idr, respectively (Yamazaki et al., 2020). Although the function of *idrP_1_P_2_* gene products is still unclear, they may be involved in detoxifying hydrogen peroxide (H_2_O_2_), which is supposed to be formed during iodate reduction (Yamazaki et al., 2020; Reyes-Umana et al., 2022a). The *idrABP_1_P_2_* gene cluster is distributed not only in marine bacteria but also in terrestrial bacteria, including subsurface and groundwater metagenome libraries, suggesting the possibility that DIRB plays a crucial role in the transformation of iodate in terrestrial environments (Yamazaki et al., 2020).

A long-lived radioactive iodine ^129^I (half-life: 1.6 × 10^7^ years) is naturally produced in the atmosphere by cosmic ray interactions with xenon, and by the spontaneous fission of ^238^U in the earth’s crust (Fabryka-Martin et al., 1985). However, in the post nuclear era, the amount of anthropogenic ^129^I released from nuclear weapon tests and nuclear facilities is higher than that produced naturally (Kaplan et al., 2014). ^129^I was also released into the environment from two major nuclear accidents, Chernobyl in 1986 and Fukushima in 2011 (Yeager et al., 2017). Due to its possible accumulation in the human thyroid gland and rapid mobility in the subsurface environment, ^129^I is one of the three key risk drivers at several US Department of Energy (DOE) sites, such as the Hanford Site and the Savannah River Site (SRS) (Kaplan et al., 2014). Both sites have contaminant plumes containing ^129^I at concentrations well above the drinking water standard of 0.04 Bq L^−1^ (Neeway et al., 2019). Iodine speciation analysis in groundwater samples recovered from the Hanford site indicated that iodate was the predominant species (Zhang et al., 2013). Similarly, in groundwater samples collected from the SRS, iodate accounted for 7 to 100% of the ^129^I (Otosaka et al., 2011). Radioactive iodine can be removed and sequestered by silver-impregnated sorbents such as zeolite and granular activated carbon, but they are ineffective at removing iodate (Kaplan et al., 2019; Li et al., 2019). Therefore, bacterial iodate reduction to form iodide could be a promising way to help the restoration of ^129^I-contaminated subsurface aquifers. However, to date, there are only scarce information on iodate reduction by terrestrial bacteria.

In this study, we focused on *Azoarcus* sp. DN11, a denitrifying benzene-degrading bacterium previously isolated from a gasoline-contaminated subsurface aquifer at Kumamoto-city, Japan (Kasai et al., 2006, 2007). Because strain DN11 is a terrestrial bacterium possessing a putative *idrABP_1_P_2_* gene cluster in its genome, we first determined whether this strain could perform iodate respiration. The involvement of the Idr proteins in iodate respiration was confirmed using proteomic and transcriptomic techniques. Finally, silver-impregnated zeolite was added to the spent medium of strain DN11 to assess its potential use in the removal and sequestration of ^129^I from contaminated aquifers.

## Results

### Iodate respiration by *Azoarcus* sp. DN11

**Figure 1:**
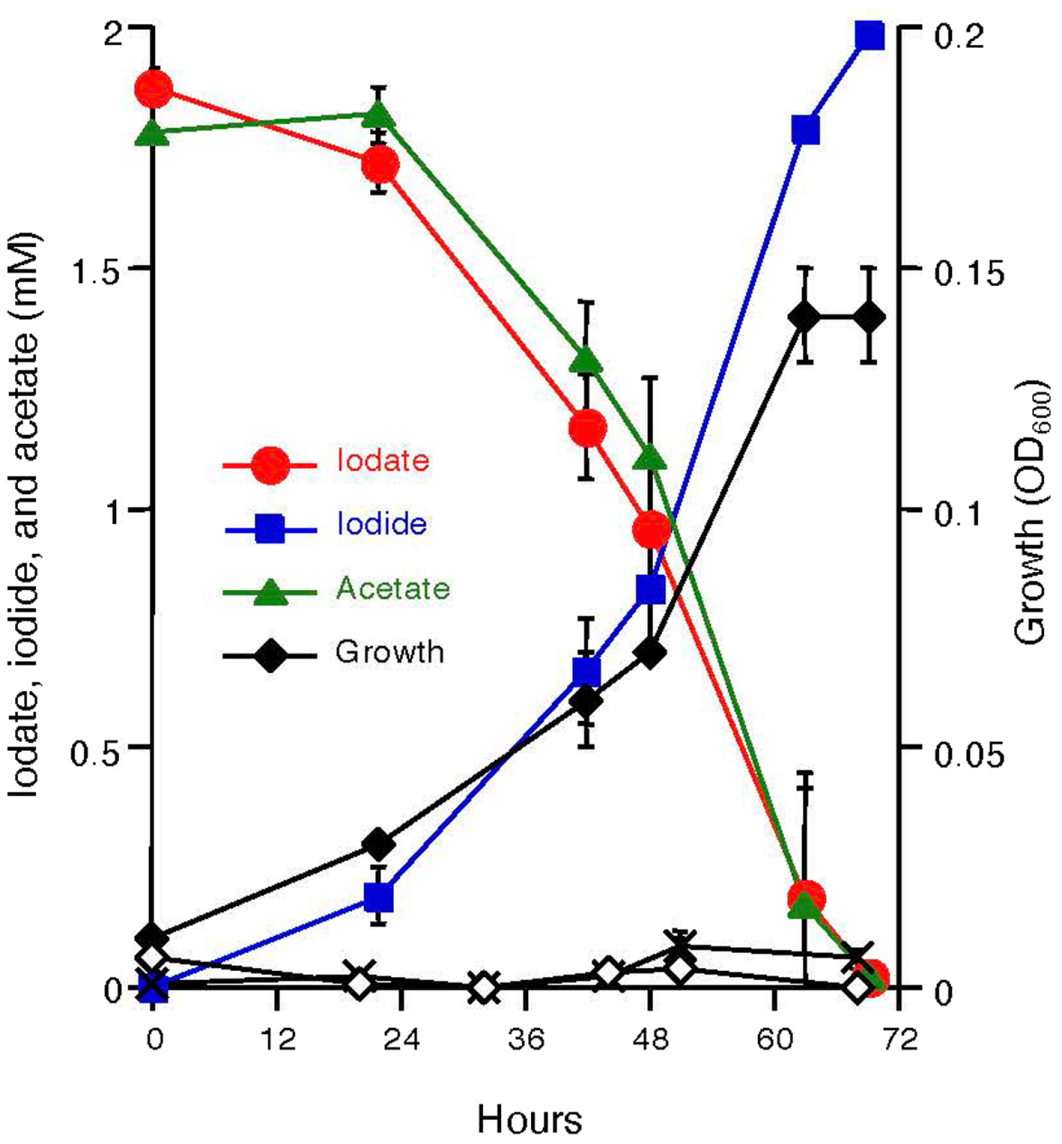
Growth of strain DN11 with 2 mM acetate as the electron donor and 2 mM iodate as the electron acceptor. Growth in the absence of acetate (white diamonds) and in the absence of iodate (crosses) is also shown. All experiments were performed in triplicate, and error bars represent standard deviations. The absence of bars indicates that the error is smaller than the symbols.

*Azoarcus* sp. DN11 was grown anaerobically using 2 mM acetate and 2 mM iodate as the electron donor and acceptor. As shown in Figure 1, strain DN11 coupled acetate oxidation to iodate reduction, and iodate was reduced entirely to an equivalent amount of iodide. The growth of strain DN11 coincided with the reduction of iodate, but the strain did not grow when iodate or acetate was omitted from the growth medium. These results strongly suggest that acetate and iodate are required for the growth of strain DN11 and that iodate reduction by this strain is a respiratory process. The following equation can express acetate oxidation coupled with iodate reduction:

3C_2_H_3_O_2_^−^ + 4IO_3_^−^ + 3H^+^ → 4I^−^ + 6CO_2_ + 6H_2_O (ΔG^0′^ = −743 kJ/mol acetate).

This indicates that 1.5 mM of acetate is required for the respiratory reduction of 2 mM iodate. However, as shown in Figure 1, nearly equal amounts of acetate and iodate were consumed by DN11. This can be explained by the incorporation of some carbon into the cell biomass.

### Activity and identification of Idr

Strain DN11 was grown in the presence of 2 mM iodate or 2 mM nitrate as the electron acceptor, and Idr activity and respiratory nitrate reductase (Nar) activity in the crude extracts were determined using reduced methyl viologen (MV) as an electron donor. Under iodate-respiring conditions, significant Idr activity (2.98 U mg protein^−1^) was observed, while no significant Nar activity (less than 0.1 U mg protein^−1^) was detected in the crude extracts (Supplementary Table S1). Under denitrification conditions, no significant Idr activity was observed, while 0.96 U mg protein^−1^ of Nar activity was detected (Supplementary Table S1). These results suggest that Idr activity is induced under iodate-respiring conditions in DN11.

**Figure 2:**
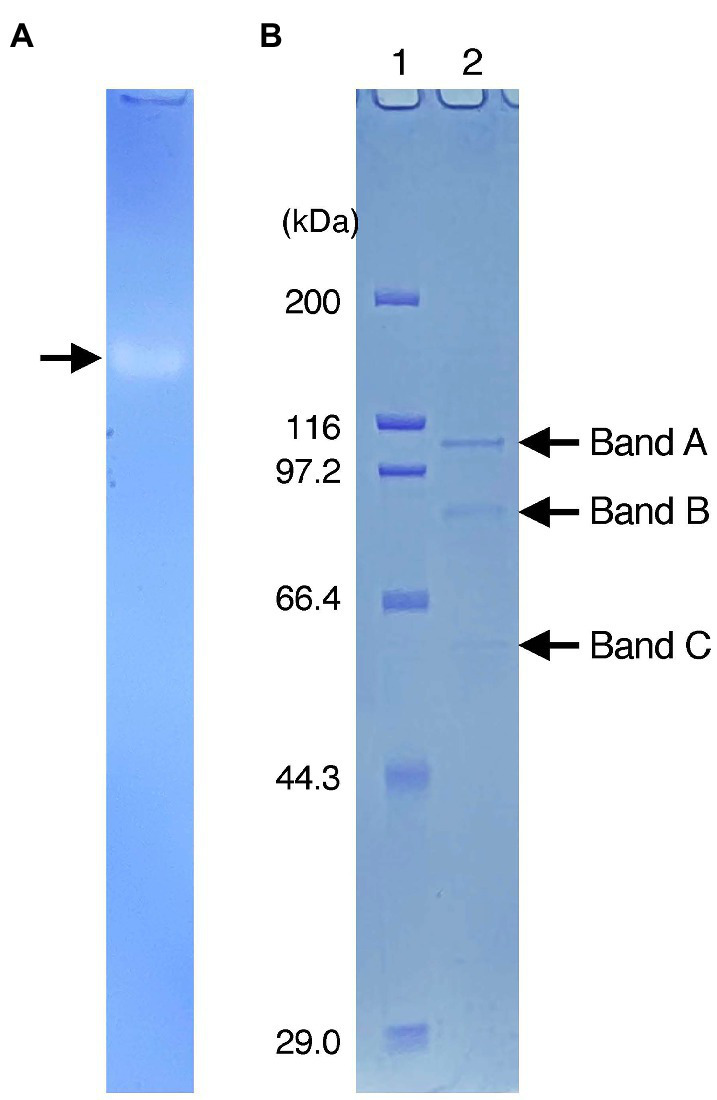
**(A)** Activity staining of Idr protein. The crude extracts were run on sodium dodecyl sulfate (SDS)-polyacrylamide gel electrophoresis at 4°C under non-denatured conditions (without SDS and reducing agents). After the electrophoresis, the gel was stained anaerobically with methyl viologen and iodate as the electron donor and acceptor, respectively. A clear band represents the active band. **(B)** Separation of the excised active band under completely denatured conditions (100°C, 5 min with SDS and 2-mercaptoethanol). The gel was stained with Coomassie brilliant blue R-250. Lane 1 represents the standard marker proteins. Three bands with molecular weights of approximately 100 kDa (band A), 80 kDa (band B), and 50 kDa (band C) were detected.

After crude extracts were subjected to SDS-polyacrylamide gel electrophoresis (SDS-PAGE) under non-denaturing conditions, the gel was stained for Idr activity. A single clear band was observed on the gel (Figure 2A), and it was then excised and run again on SDS-PAGE under fully denaturing conditions. As shown in Figure 2B, three bands (designated A, B, and C) with apparent molecular weights of 100, 80, and 50 kDa were observed after Coomassie brilliant blue (CBB) staining. All bands were excised, trypsin-digested, and subjected to liquid chromatography–tandem mass spectrometry (LC–MS/MS) analysis. From band A, 49 proteins with apparent molecular weights of 16–171 kDa were identified (Supplementary Table S2A). Among these, a protein with accession number AYH44092.1 showed the highest sequence coverage of 77%. In addition, this protein had the highest exclusive unique peptide count of 72. BLASTP analysis of this protein revealed that it is a homolog of IdrA in *Pseudomonas* sp. SCT. Other proteins included homologs of IdrP_1_ (AYH44094.1) and IdrP_2_ (AYH44095.1), with a sequence coverage of 15 and 20%, respectively. From band B, 56 proteins were identified, among which the homologs of IdrA, IdrP_1_, and IdrP_2_ were detected with sequence coverages of 31, 41, and 39%, respectively (Supplementary Table S2B). Similarly, 88 proteins were identified from band C, from which the homologs of IdrA, IdrP_1_ and IdrP_2_ were detected with sequence coverage of 45, 19, and 29%, respectively (Supplementary Table S2C). The fact that homologs of IdrA, IdrP_1_ and IdrP_2_ were detected repeatedly from bands A, B, and C with relatively high sequence coverage strongly suggests that these proteins are involved in iodate respiration by strain DN11.

### Organization of *idr* gene cluster in strain DN11

In the genome of DN11, the *idrABP_1_P_2_* gene cluster formed an operon-like structure (Supplementary Figure S1A). Upstream of the *idr* gene cluster, a putative sigma-54 dependent transcriptional regulator gene (*atoC*) and a two-component system-sensor histidine kinase gene (*atoS*) were located. However, *c*-type cytochrome gene as well as molybdenum cofactor biosynthesis genes, both of which are present in the vicinity of the *idr* gene cluster in *Pseudomonas* sp. SCT (Yamazaki et al., 2020), were absent. One exception was that the *moeB* gene was upstream of the *atoS* gene. As shown in Supplementary Figure S1B, the amino acid sequence of IdrA (AYH44092.1) predicted that it included a [3Fe-4S]-binding motif (CX_2_CX_3_CX_70_S) and molybdopterin-guanine dinucleotide coordination motif. In addition, IdrB (AYH44093.1) appears to include a [2Fe-2S]-binding motif (CXHX_18_CX_2_H) and a twin-arginine translocation (TAT) signal sequence (SRRSFL) at the N-terminal region. Both IdrP_1_ (AYH44094.1) and IdrP_2_ (AYH44095.1) are diheme proteins containing two heme-binding motifs (CX_2_CH). The SignalP 5.0 server predicted that both IdrP_1_ and IdrP_2_ possessed a signal peptide sequence for the Sec translocation system. These features were similar to those observed for IdrABP_1_P_2_ of *Pseudomonas* sp. SCT (Yamazaki et al., 2020), and suggest that these Idr proteins are secreted and function in the bacterial periplasmic space.

### Transcriptional analysis of *idr* genes

The expression of *idrA*, *idrP_1_*, and *idrP_2_* relative to the expression of *recA* was quantified and compared using RNA extracted from iodate-, nitrate-, or aerobically (O_2_)-grown cells of strain DN11. The respiratory nitrate reductase gene (*narG*) expression was also quantified. As shown in Table 1, the *idrA* gene in iodate-grown cells showed 104- and 59-fold upregulation compared to that in nitrate- and O_2_-grown cells, respectively (0.71 versus 0.0068 versus 0.012). Similarly, *idrP_1_* expression in iodate-grown cells was 53- and 67-fold higher than in nitrate- and O_2_-grown cells. The expression patterns of *idrP_2_* were similar to those of *idrA* and *idrP_1_*; 26- and 12-fold upregulation was observed in iodate-grown cells. In contrast, the expression of *narG* was highest in nitrate-grown cells, which was 1,260-fold higher than that in iodate- and O_2_-grown cells (2.4 versus 0.0019).

**Table 1:** Expression of *idr* genes and *narG* gene relative to that of *recA* gene in iodate-, nitrate-, or aerobically grown cells.

Gene	Growth condition		
	Iodate	Nitrate	O_2_ (aerobic)
*idrA*	0.71 ± 0.019^a^	0.0068 ± 0.0013	0.012 ± 0.0016
*idrP_1_*	1.0 ± 0.030	0.019 ± 0.0058	0.015 ± 0.0019
*idrP_2_*	2.5 ± 0.10	0.097 ± 0.032	0.21 ± 0.0030
*narG*	0.0019 ± 0.00028	2.4 ± 0.019	0.0019 ± 0.00018

a Values represent the ratio of the relative quantity of *idrA*, *idrP_1_*, *idrP_2_*, or *narG* transcripts to that of *recA* gene transcripts. Data are means ± standard deviations (*n* = 3).

### Iodine removal by silver-impregnated zeolite

Strain DN11 was grown anaerobically using 200 μM iodate as the electron acceptor. After growth, the liquid culture was mixed vigorously with silver-impregnated zeolite (AgIZ), and the iodine (iodate and iodide) in the supernatant was determined. Before the inoculation of strain DN11, the iodate concentration in the medium was constant (192–193 μM), regardless of AgIZ addition (Table 2). This indicates that AgIZ did not remove iodate under our experimental conditions. However, after the growth of strain DN11, the iodide produced by strain DN11 (198 μM) was removed completely from the supernatant by AgIZ. Consequently, neither iodate nor iodide was detected in the supernatant. Because the detection limit of iodide under our experimental conditions was 5 μM, the efficacy of iodine removal was calculated to be more than 97.5%.

**Table 2:** Removal of iodine from the growth medium of strain DN11 by silver-impregnated zeolite^a^.

	Without AgIZ^b^		With AgIZ	
	Iodate	Iodide	Iodate	Iodide
Before inoculation of DN11	192 ± 1.57	<5.00^c^	193 ± 1.40	<5.00
After the growth of DN11	<3.00^d^	198 ± 3.05	<3.00	<5.00

a All values are expressed as the means of iodine concentration (μM) ± standard deviations of the data from triplicate determinations.

b Silver-impregnated zeolite.

c Less than the detection limit of 5 μM.

d Less than the detection limit of 3 μM.

## Discussion

The first DIRB, *Pseudomonas* sp. SCT, was isolated from the marine sediment of Sagami Bay (Amachi et al., 2005), and proteomic and transcriptomic analyses of this strain have suggested the involvement of IdrABP_1_P_2_ proteins in iodate respiration (Yamazaki et al., 2020). Reyes-Umana et al. (2022a) isolated another DIRB, *Denitromonas* sp. IR-12, from San Francisco Bay estuarine sediment and demonstrated that the *idrA* gene is responsible for iodate respiration by constructing a gene knockout mutant. Very recently, Reyes-Umana et al. (2022b) isolated a third DIRB from freshwater creek sediment located in the vicinity of the Northern Pacific Ocean. This bacterium, *Aromatoleum toluclasticum* TC-10, is phylogenetically closely related to *Azoarcus* sp. DN11 and coupled acetate oxidation to iodate reduction to conserve the energy for growth. In this study, we found that *Azoarcus* sp. DN11 can also grow anaerobically in the presence of acetate and iodate as the electron donor and acceptor, respectively. Strain DN11 was previously isolated from gasoline-contaminated groundwater in Higashino, Kumamoto-city, Japan (Takahata et al., 2006), approximately 15 km inland from the coastal area. To our knowledge, this is the first report of a DIRB isolated from a terrestrial groundwater system. Such bacteria could potentially be helpful for the removal and sequestration of ^129^I, which contaminates underground aquifers at relatively low concentrations (Kaplan et al., 2014).

Strain DN11 can utilize nitrate as the sole electron acceptor *via nar* genes encoding respiratory nitrate reductase (Kasai et al., 2006, 2007). However, DN11 cells grown under denitrification conditions showed no Idr activity, whereas cells grown under iodate-respiring conditions showed no Nar activity (Supplementary Table S1). Similarly, the expression of *idr* genes was downregulated under denitrification conditions, whereas *narG* was downregulated under iodate-respiring conditions (Table 1). These results were in good agreement with those observed for *Pseudomonas* sp. SCT (Yamazaki et al., 2020), and support the idea that nitrate reductase is not involved in iodate reduction by DIRB (Amachi et al., 2005; Mok et al., 2018; Reyes-Umana et al., 2022b). If strain DN11 is used for the bioaugmentation of ^129^I-contaminated groundwater in the future, pre-cultivation of this strain in the presence of stable iodate (^127^IO_3_^−^) may be necessary for sufficient induction of the *idr* gene cluster.

Previously, the active Idr band of *Pseudomonas* sp. SCT was excised and subjected to SDS-PAGE (Yamazaki et al., 2020). Three bands with molecular weights of 100, 40, and 35 kDa were observed, and IdrA, IdrP_1_, and IdrP_2_ were detected from these bands with the highest sequence coverage. This was reasonable because the predicted molecular weights of IdrA, IdrP_1_, and IdrP_2_ are approximately 100, 40, and 40 kDa, respectively. In addition, multiple Idr proteins were detected in these bands, with relatively lower sequence coverage. For example, the 100- and 35-kDa bands included all Idr proteins (IdrA, IdrB, IdrP_1_, and IdrP_2_), whereas no other proteins (non-Idr proteins) were recovered from these bands. In this study, three bands were observed after SDS-PAGE, with molecular weights of 100, 80, and 50 kDa (Figure 2). All these bands included IdrA, IdrP_1_, and IdrP_2_ proteins, but a wide variety of non-Idr proteins that are irrelevant to iodate respiration were also recovered (Supplementary Table S2). Although active bands often include proteins that are not targeted or differ from the predicted molecular weight, the exact reason for this is still unclear (Tsuchiya et al., 2019; Muramatsu et al., 2020). Nevertheless, the fact that active bands recovered from both strains SCT and DN11 included multiple Idr proteins strongly suggests that IdrABP_1_P_2_ forms a protein complex possibly in the bacterial periplasmic space. Further biochemical studies are required to understand the molecular weight and subunit composition of the Idr protein complex.

Yamazaki et al. (2020) proposed a hypothetical model for iodate respiration by *Pseudomonas* sp. SCT, in which iodate is first reduced to an intermediate, hypoiodous acid (HIO), by IdrAB with concomitant hydrogen peroxide (H_2_O_2_) production. H_2_O_2_ is then reduced to H_2_O by IdrP_1_ and (or) IdrP_2_ using reduced cytochrome *c* as the electron donor. HIO, a potentially toxic compound, is assumed to be detoxified by a chlorite dismutase (Cld)-like protein, which is abundantly expressed in iodate-respiring cells of strain SCT, to form iodide and molecular oxygen.


$$\mathrm{HIO}\to H^{+}+I^{-}+1/2\mathrm{O2}$$

Reyes-Umana et al. (2022a) proposed an alternative model of iodate respiration in which HIO is chemically disproportionated to yield iodide and iodate in a 2:1 ratio since HIO is thermodynamically unstable, especially at pH > 5.0.


$$\mathrm{3HIO}\to {\mathrm{IO}}_{3}{}^{-}+{\mathrm{2I}}^{-}+\mathrm{3H+}$$

Although whether HIO is degraded biochemically or abiotically in DIRB is controversial, we could not find a gene encoding a Cld-like protein in the draft genome of strain DN11. This might indicate that HIO is degraded abiotically or that a yet-identified protein is involved in HIO degradation in strain DN11.

Combining DN11 and AgIZ, iodate was removed completely from the aqueous culture medium as solid silver iodide (AgI) (Table 2). Due to ion chromatography’s high detection limits (3 μM for iodate and 5 μM for iodide), we applied 200 μM iodate, which is several orders of magnitude higher than the environmentally relevant concentration. The average total iodine concentration in groundwater collected from the Hanford Site was approximately 0.2 μM, 45–84% as iodate (Zhang et al., 2013). In SRS F-area, on the other hand, iodate concentration was reported to be ranging from 0 to 0.075 μM (Otosaka et al., 2011). Since the solubility product of AgI is very low (*K*_sp_ = 1 × 10^−16^), more than 3 × 10^−5^ μM of iodide can theoretically be removed by AgIZ as AgI. Thus, the possible limiting factor for the restoration of ^129^I in subsurface aquifers might be the affinity of Idr for its substrate iodate but not the effectiveness of AgIZ. A detailed biochemical study of Idr is required to elucidate its kinetics (*K_m_*, *V*_max_, and *k*_cat_) toward iodate. Moreover, further study is needed to understand the effect of other competitive electron acceptors such as nitrate on iodate reduction by strain DN11. Adsorption of iodate to Fe- and Al-containing minerals (Yoshida et al., 1992) may also affect bacterial iodate reduction in terrestrial environments.

## Materials and methods

### Growth conditions and culture media

*Azoarcus* sp. DN11 was previously isolated from gasoline-contaminated groundwater in Kumamoto City, Japan (Kasai et al., 2006, 2007). The strain was routinely grown anaerobically at 30°C in the basal medium, as described previously (Yamazaki et al., 2020). One liter of the medium contained NH_4_Cl (0.54 g), KH_2_PO_4_ (0.14 g), MgCl_2_·6H_2_O (0.20 g), CaCl_2_·2H_2_O (0.15 g), Na_2_SO_4_ (0.14 g), casamino acids (0.10 g), NaHCO_3_ (2.5 g), a vitamin solution (1.0 mL), and a trace metal solution (1.0 mL). Acetate (2 mM) and iodate (2 mM) were added to the medium from the sterile anaerobic stock solutions. The medium (20 mL) was dispensed into a 60 mL serum bottle under an N_2_-CO_2_ (80:20) gas stream, and the bottle was sealed with a thick butyl rubber stopper and an aluminum cap. In some cases, nitrate (2 mM) or oxygen was used as the electron acceptor, as previously described (Yamazaki et al., 2020).

### Preparation of crude cell extracts and enzyme assays

To prepare crude extracts, cells grown on iodate or nitrate for 48 h were collected, washed, and resuspended in 10 mM potassium phosphate buffer (pH 7.0). The cells were disrupted by sonication (Q500 Sonicator; Qsonica, Newtown, CT, United States), followed by centrifugation (17,000 × *g*, 30 min, 4°C) to remove cell debris, and the supernatant was used as the crude enzyme. As described previously, the reductase activity was assayed by a spectrophotometer (Yamazaki et al., 2020). The reaction mixture contained 20 mM Tris–HCl (pH 6.8), 0.3 mM MV (ε_578_ = 9.7 mM^−1^·cm^−1^), an appropriate amount of enzyme, and an electron acceptor (iodate or nitrate). After the reaction mixture was degassed and sparged with N_2_ gas, the reaction was initiated by adding sodium dithionite. One unit (U) of reductase activity was defined as the enzyme required to oxidize 1 μmol of reduced MV per minute. Protein concentration was determined using a protein assay kit (Bio-Rad, Hercules, CA, United States), with bovine serum albumin as a standard protein.

### Electrophoresis, activity staining, and LC–MS/MS analyses

Electrophoresis was performed in two steps to separate the proteins included in the crude extracts. In the first step, non-denaturing gel electrophoresis was performed at 4°C using a 10% polyacrylamide gel in 25 mM Tris-glycine buffer (pH 8.3), omitting both reducing agents and SDS. Following non-denaturing electrophoresis, the gel was incubated in a nitrogen atmosphere with 20 mM Tris–HCl (pH 6.8) containing 0.3 mM MV, 12 mM iodate, and 10 mM dithionite. In the second step, the clear band (active band) that appeared on the gel was excised, boiled for complete denaturation (with 2% SDS and 5% 2-mercaptoethanol for 5 min), and then subjected to SDS-PAGE again. The Protein Molecular Weight Marker (Takara, Otsu, Japan) was used as the standard marker. Proteins were visualized by staining with CBB R-250, and the selected bands were excised, trypsin-digested, and subjected to LC–MS/MS analysis, as described previously (Shevchenko et al., 1996).

### Transcriptional analysis

All manipulations were performed as previously described (Yamazaki et al., 2020). RNA was prepared from cultures under all growth conditions, i.e., on iodate, nitrate, or oxygen as the terminal electron acceptor. Cells in the late exponential growth phase were collected, and pellets were stored at −80°C. Total RNA was extracted using the RNeasy Miniprep kit (QIAGEN, Hilden, Germany), followed by treatment with a TURBO DNA-free kit (Ambion, Carlsbad, CA, USA) to remove residual DNA. cDNA was synthesized using a SuperScript First-Strand Synthesis System for RT-PCR (Invitrogen, Carlsbad, CA, USA). Quantifying *idrA*, *idrP_1_*, *idrP_2_*, and *narG* in the cDNA samples was performed using quantitative PCR (qPCR). The *recA* gene was quantified in the cDNA samples to normalize the qRT-PCR data. As shown in Supplementary Table S3, new primers were designed for qPCR analysis. SYBR green detection chemistry was used for qPCR assays using a Step One Plus instrument (Applied Biosystems). Standard curves were constructed using serial dilutions of cDNA prepared from cells grown on iodate. The slopes of the standard curves were used to calculate PCR efficiencies according to the following equation:


$$E\;\left(\%\right)=\left[10^{\left(-1/\mathrm{slope}\right)}-1\right]\times 100$$

The *E* values obtained for all genes and primer pairs ranged from 90 to 110%, with *R*^2^ values greater than 0.98.

### Iodine removal by silver-impregnated zeolite

Strain DN11 was grown anaerobically in a basal medium containing 200 μM iodate as the electron acceptor. After 3 days of growth, 1 mL of the liquid culture was removed and mixed with 0.2 g of silver-impregnated zeolite (NE-AG/ZEO-KUR, Nuclear Engineering Co., Ltd., Ibaraki, Japan). After vigorous shaking, the mixture of liquid culture and zeolite was centrifuged (15,000 × *g*, 3 min, 4°C) to remove the cells and zeolite. Iodate and iodide in the supernatant were determined using ion chromatography (see below).

### Analytical techniques

The concentrations of iodate, iodide, and acetate were determined using an ion chromatograph IC-2010 (Tosoh, Tokyo, Japan), as described previously (Yamazaki et al., 2020). The detection limits of iodate and iodide were 3 μM and 5 μM, respectively.

## Data availability statement

The datasets presented in this study can be found in online repositories. The names of the repository/repositories and accession number(s) can be found at: https://www.ncbi.nlm.nih.gov/genbank/, CP021731.1.

## Author contributions

SA designed the research and acquired funding. SS and SA performed the experiments, wrote the draft manuscript, and created the figures and tables with guidance from TO and NK. All authors contributed to the data analysis, reviewed the manuscript, and approved the submitted version.

## Funding

This study was financially supported by JSPS KAKENHI (grant number 20H02896).

## Conflict of interest

The authors declare that the research was conducted in the absence of any commercial or financial relationships that could be construed as a potential conflict of interest.

## Publisher’s note

All claims expressed in this article are solely those of the authors and do not necessarily represent those of their affiliated organizations, or those of the publisher, the editors and the reviewers. Any product that may be evaluated in this article, or claim that may be made by its manufacturer, is not guaranteed or endorsed by the publisher.
