# Supplementary material for: Iodate respiration by Azoarcus sp. DN11 and its potential use for removal of radioiodine from contaminated aquifers
Source: Front Microbiol. 2023 Apr 17;14:1162788. doi: 10.3389/fmicb.2023.1162788 (PMC10149662; doi:10.3389/fmicb.2023.1162788)
Supplement: Supplementary file 1 [file Data_Sheet_1.pdf]

Table S1. The Idr and Nar activities in the crude extracts of strain DN11 grown with iodate or nitrate[a].

Cells grown with	Idr activity	Nar activity
Iodate	2.98 ± 0.37	<0.100[b]
Nitrate	<0.100	0.96 ± 0.12

[a] *Idr and Nar activities are expressed as the means of specific activity (µmol of MV oxidized per minute per mg of protein) ± standard deviations of the data from triplicate determinations.*

[b] *The activity was below the detection limit of 0.100 U per mg of protein.*

Table S3. Primers used for qRT-PCR assays

**Primer**	**Sequences (5' to 3')**
*idrA*_2070f	GCTTACCGCCTTCAACACC
*idrA*_2159r	ACCGCCTCGTAGAACTGGA
$idrP_1$_863f	GCAAGTTCCGCACACCATC
$idrP_1$_970r	CGCCCGCATCGTAGAAA
$idrP_2$_13f	AATGGCACAGGGTTTGCAG
$idrP_2$_98r	CGCGTGTTGTTCGTCGTT
*narG*_2230f	AAGGGCACGAAGGAAGTGAA
*narG*_2344r	GCAGGATGATGTCGGAGTAGG
*recA*_459f	CGAAATCGAAGGCGAGATG
*recA*_560r	GTGTTGGTGCGCTTGATGTT

A

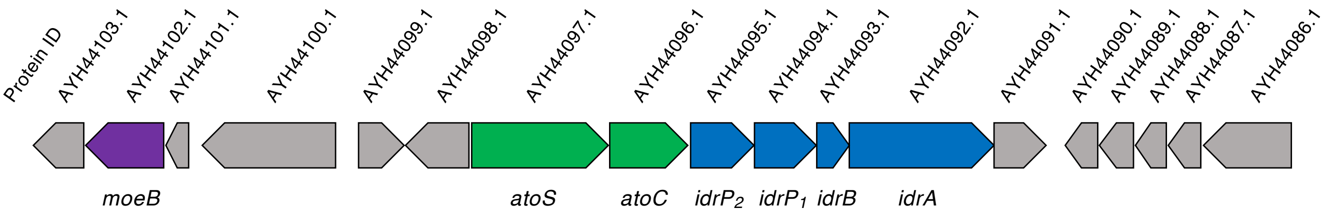

B

>AYH44092.1 iodate reductase large subunit [*Azoarcus* sp. DN11]
MSIHDKNTAKDFVPLPPKDADVFTTACDYCTVACGYKVYRWPVGREGGAKAAQNALRADFPHQMM
MSAWVSPSQHNVVSFKGKPHHVVVVPDKDATVVNVGGNHSIRGGTLAEKCYNPDNLTRERLQHPMI
RVNGRLTPVTWDLATEVMADISQYILTKYGEHAWGMKTYSYQYFENTYAITKLAMTSIGTPAFAWHDK
ASNTNDATGLDDAGIDSFASSYQDWADCEVAFLSGVDPYETKTTLFTTWMMPGDKKFIFVTPHKTTG
VAWSLREGRGLWLPVIPGTDTVLHMALARIIIENNWQDQEFIDKWIANSWEVDSGYGRGTRNTGWQ
WRTTWGTWQSDWPDYKKFILSQEESKLDVAAKITGLDPADIVKAAEWIAKRKADGSLPKTSFMCEKG
NYWSNNYMNTTSFASLGLICGAGNRPGRMISRGGGHQRGGLVAGGGSSWLSPEKYPGRRKKSFNL
DLWLMEGNLRFAWVIGTTWIAAMMGSNALEKRMRELTVDSPHQITHLDRRSIFETLKRRVDGGGMV
MVNSDIYPVMPIGTELADIVLPAATWGEDNFTRCNSERRLRLYSKFYDAPGEAKPDWWIIQKFAQKMG
LDKDGGYNWKDSNDVFEEGSRYSRNGVLSYHVLTDEAKTKGVKAQELLRTYGTTGIQTPIRKRDGKL
VGTQRLHDPTNDWGELEGVTVQTKVLTAFNTQSGKAILLKSPWKYAGWIQFYEAVKPRAEKGELWVT
NGRVNETWQSGFDDLRKPYLSQRWPEAFLFIHPDDAAKRGIESGDYVEVVNDTVYVQTGQPQGVLD
ADLNFNDLMRDGHIMTTVGRFRTVAIVSDEMRPGVTKANFNFPKSPANAVVSAVPDPMTNNYRYKLG
RGVVSKVGESPYKHEFARMSLKPRNIV

>AYH44093.1 iodate reductase small subunit [*Azoarcus* sp. DN11]
MSQTSSQRAIQIRQPAGHDHDHEGGRTCMSRRSFLLTGGAALALVSLGGIPGFAEAAQLLKASYARQ
KIGSLSALKVGEPLTFNYPYADVRNILVKLGAPAGGGIGPDSDIVAFNQQCTHMGGPLDGTYKPGHQV
LGPCPLHLTSFDLTRHGMVVAGHATESLPQITLEVQGDDIYAVGVMGLVYGYSAMTAEHRA

>AYH44094.1 cytochrome-c peroxidase [*Azoarcus* sp. DN11]
MKNRTTLKLSAIAASFAVVLTATAATGQTFPPLAPLPPVPSPKDNPQSPEKIALGKQLFWDSRLSGDG
SMPCVSCHLPALGWGDNNAISRGYPGTQHWRNSQTILNAAYYNKLFWDGTSTSLESEASSAAEGSV
AGNGDPSVMEMRMRFLPEYVEAFKKVFGAEWPRMNDAYRAISSYQRTVVSDASKVPFDRYANGDR
KALNDAQKRGMTLFNGKAGCIQCHNGPLASDQKFYNLGLPDFEGFKTDVLYQVTHRWEHYQKGVAE
QKYRSADMDYGLYFQTKNPKDIGKFRTPSLRELKYTAPYMHNGVFKTLEQVVDFYDAGGGNAANKS
ELLKPLGLTAQEKADLLAF LDALSMSEPLIHDEPKLPGAYQPLPAPTK

>AYH44095.1 cytochrome-c peroxidase [*Azoarcus* sp. DN11]
MCHENGTGFAVPLAIVGSFFVQGEIMKTTNNTRTGRLLAACSMAAAAWLLPSGAQGAAPDLAPMPEV
KPGNAAMIELGKYFFFDRRLSGDWGRSCASCHDPAKGWGDGQALSTGYPSMEYFRNAPTVLNAKY
RQRFMWDGRLDGTDAGTLVRDMITETHTMNMDGRLMQERLKQVPEYDALWKKWRNDDINGMRVF
NVIGEFIRSLETTNAPFDKFARGDADAISAEAKEGYALFKGKAGCVACHNGPIGSDGKLYKTGVPEHP
DVLANPLRTITMLRHYATNGLPNYMSARTDVGAYAITKNPADIGKFQTAQLRDLKYTAPYMHNGVFAT
LDEVVAFYNRGGGEGSGLKPLNLSAGEQKALVIFLLTLSGDPVVVANPGQPDMQVRTFGKN

Figure S1. (A) The putative *idr* operon (locus tag AYH44092.1 to AYH44097.1) found in the draft genome of *Azoarcus* sp. DN11 (CP021731.1), which consists of *idr* genes (blue) and genes encoding putative transcriptional regulatory proteins (green). A molybdenum cofactor biosynthesis gene *moeB* (purple) is also shown. (B) Structural motifs predicted in putative IdrA (AYH44092.1), IdrB (AYH44093), IdrP$_1$ (AYH44094.1), and IdrP$_2$ (AYH44095.1). For IdrA, the putative [3Fe-4S]-binding motif and the cysteine residue predicted to coordinate the guanine dinucleotide molybdopterin cofactor are highlighted in pink and cyan, respectively. For IdrB, the putative twin-arginine translocation motif and the predicted [2Fe-2S]-binding motif are highlighted in green and yellow, respectively. For IdrP$_1$ and IdrP$_2$, two heme-binding motifs are highlighted in red.
